# Numerical Validation of Two-Parameter Weibull Model for Assessing Failure Fatigue Lives of Laminated Cementitious Composites—Comparative Assessment of Modeling Approaches

**DOI:** 10.3390/ma12010110

**Published:** 2018-12-31

**Authors:** Asad Hanif, Yongjae Kim, Cheolwoo Park

**Affiliations:** 1 Department of Civil Engineering, Mirpur University of Science and Technology (MUST), Allama Iqbal Road Mirpur AJ&K, Mirpur 10250, Pakistan; ahanif@connect.ust.hk; 2 Department of Civil Engineering, Kangwon National University, 346, Jungang-ro, Samcheok-si 25913, Gangwon-do, Korea

**Keywords:** flexural fatigue, laminated composite, cementitious composite, ProFatigue, modeling

## Abstract

In this paper, comparative assessment of failure fatigue lives of thin laminated cementitious composites (LCCs) modeled by two modeling approaches—double-parameter Weibull distribution model and triple-parameter distribution model—was carried out. LCCs were fabricated of ordinary Portland cement (OPC), fly ash cenosphere (FAC), quartz sand, and reinforcing meshes and fibers. The failure fatigue life assessment at various probabilities by the two-parameter model was based on numerical calculations whereas the three-parameter model was applied by an open source program—ProFatigue^®^. Respective parameters, shape and scale parameters in the two-parameter Weibull distribution model while shape, scale, and location parameters in three-parameter model were determined, and the corresponding probabilistic fatigue lives at various failure probabilities were calculated. It is concluded that the two-parameter model is more accurate in probabilistic fatigue life assessment of double-layer mesh-reinforced LCCs, whereas for single-layer reinforced LCCs, both models could be used at a fair confidence level.

## 1. Introduction

Thin laminated cementitious composites (LCCs) are special thin-walled reinforced concrete elements constructed of cement mortar reinforcement meshes [1] offering significant advantages over cement-based composites, such as convenient manufacturing, cheaper production, improved flexural characteristics, higher crack resistance, and toughness. Thus, they are well suited for structural retrofitting and construction of water retaining structures (pools and tanks), domes/shells, and partitions in buildings [2,3]. To attain the optimum benefits of LCCs, fabrication of structural elements with greater strength to unit weight ratio is highly imperative, which is why many research findings published earlier focused the development of lightweight LCCs on improved flexure and toughness attributes [4,5,6,7,8,9].

While the structural lightweight LCCs have been successfully developed and evaluated for excellent mechanical and flexural performance [10], the fatigue characteristics and the corresponding modeling for assessing the probabilistic fatigue damage were not appropriately addressed earlier. As a result, the knowledge on structural behavior of LCCs has gained maturity, whereas the long-term performance (fatigue) has remained not well-known.

Previously much work has been carried out on the fatigue life assessment and modeling of cement-based composites including plain concrete [11,12,13,14,15], steel fiber-reinforced concrete [16,17,18,19,20,21], PVA fiber-reinforced concrete [22,23], recycled aggregate concrete [16,17,18,19,20,21], and ferrocement and thin laminated composites [24,25]. In recent years the work of Huang et al. [26,27,28,29,30] is worth mentioning. In these published findings, various properties were attempted to be modeled such as compression [26,27,29], flexure [31,32], and tension [33]. The fatigue modeling (for compression, tension, and failure) conducted on cement-based composites has primarily used the two-parameter model approach for evaluating the failure fatigue lives and likelihood of failure under diverse dynamic loadings. The two-parameter model approach involves determination of two parameters, viz shape parameter (α) and scale parameter (u). Surprisingly, studies on any other modeling approach, which could have yielded potentially more accurate prediction models, are sparse. Further, specific studies concerning the fatigue modeling of LCCs and ferrocement are missing.

In the current study, the three-parameter model, involving the three parameters namely shape parameter, scale parameter, and location parameter, was applied to predict the failure fatigue behavior of lightweight LCCs by using an open source software ProFatigue^®^ which has been successfully employed previously in other research findings [34,35,36]. The motivation behind the study was to comparatively evaluate the three-parameter Weibull model for better assessment of failure fatigue lives under various probabilities for cementitious composites, more specifically for LCCs. The anticipated better modeling approach could not only help predict the failure life of cementitious composites, but also ease the calculation process by using ProFatigue^®^.

## 2. Experimental Work

### 2.1. Fabrication of LCC Specimens

LCCs were fabricated by incorporating various fly ash cenosphere (FAC) weight fractions (40%, 50%, and 60%) while sand containing mortar matrix specimens was also casted for comparison. The mixing and casting method was already explained earlier in another study [4,37] while the detailed mix proportions of the matrices were given already in ref. [25]. The preparation of the mixes was done in a 20 L Hobart mixer The powders were dry mixed for a minute followed by the addition of half of the total required water while continuously mixing for another 30 s. Then, the remainder water and super-plasticizer were added with the mixing continued for additional 30 s. Later, the poly-vinyl alcohol (PVA) fibers were gradually dispersed in the mix and the mixing continued for another three minutes, until mix uniform consistency and cohesiveness were achieved. The whole mixing procedure took 6–8 min for each mix. The mortar mixes were then cast into pre-lubricated steel molds [25]. Rectangular specimens (350 mm × 100 mm × 20 mm) reinforced with Galvanized iron (GI) wire mesh, fiber-glass mesh and PVA fibers (Table 1 and Table 2) were fabricated.

### 2.2. Fatigue Test Program

**Figure 1:**
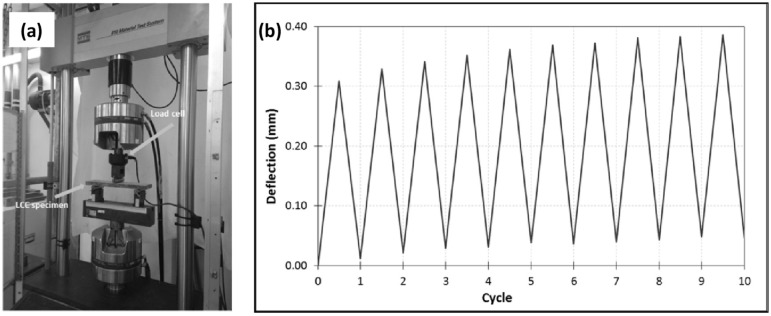
(**a**) Test for flexural fatigue, and (**b**) Studied loading in fatigue tests [25].

The fatigue lives at various stress levels of the LCCs were determined by subjecting the LCC specimens (MTS810 Test System, as shown in Figure 1; support span 300 mm, loading span 100 mm, and LVDTs for deformation measurement) under load control mechanism between two limits (with a sinusoidal force variation in time) at different stress levels ‘*S*’ (*S* = *f*_max_/*f_r_*, where *f*_max_ = maximum fatigue stress, and *f_r_* = static flexural stress).

The minimum stress level, ‘*f*_min_’, is 10% of the monotonic strength and the maximum stress level, ‘*f*_max_’, ranges from 60% to 90% of the monotonic strength. The minimum stress level, ‘*f*_min_’, was kept constant at 0.10 throughout the investigation. With constant amplitude, sinusoidal loads were applied at a frequency of 1.5 Hz and the corresponding data acquired at 0.30 s intervals. The number of cycles to failure for the specimens under different load conditions was noted as fatigue life ‘N’. With a decrease in the stress level, the number of cycles to failure of the specimens was increased. As fatigue testing is a time taking and expensive process, and a large number of samples were proposed to be tested, an upper limit on the number of cycles to be applied was selected as 2 million cycles [31]. The test was ended when the failure of the specimen occurred or the upper limit was reached, whichever occurred earlier. Table 3 indicates the cycles to failure at various stress levels.

## 3. Fatigue Modeling Approaches for Cementitious Composites

### 3.1. Two-Parameter Weibull Distribution Model

The flexural fatigue modeling done by the famous two-parameter Weibull distribution model uses the S-N relationship, given in (1) [14,16,31,38,39,40]

(1) $$S=\frac{f\max}{fr}=a+b\log10(N)$$

where, ‘*S*’ refers to the stress range, ‘*f*_max_’ is the maximum stress level to which the specimen is subjected, ‘*N*’ is the number of cycles to failure, and ‘*a*’ & ‘*b*’ are experimental coefficients. The experimental coefficients (‘*a*’ and ‘*b*’) of Equation (1) can be obtained for LCCs with different reinforcement ratios, and different FAC weight fractions from the fatigue test data obtained in this investigation using regression analysis (based on method of least squares) by plotting the stress level against log_10_(*N*). The values of material coefficients as obtained from the regression analysis are *a* = 1.1808 and *b* = −0.0924 for single layer FG reinforced LCC, *a* = 1.1280 and *b* = −0.0844 for single layer GI (Galvanized Iron) reinforced LCC, *a* = 1.1352 and *b* = −0.0903 for double layer FG (Fiber Glass) reinforced LCC, and *a* = 1.1700 and *b* = −0.0957 for double layer GI reinforced LCC.

Then, the shape parameter (*α*) and scale parameter (*u*) are determined, either by Graphical Method, Maximum Likelihood Approach, or Method of Moments. Method of moments (Equations (2) and (3)) was chosen here as it is easier to determine the required parameters. Procedural details are already covered in the literature [40,41].

(2) $$\alpha ={\left(COV\right)}^{-1.08}$$

(3) $$u=\frac{\mu }{\Gamma \left(\frac{1}{\alpha }+1\right)}$$

where *µ* is the sample mean of the fatigue-life data at a given stress level; COV (= σ/*µ*, σ is standard deviation of sample) is the coefficient of variation of the data; and Γ ( ) is the gamma function.

Based on the S-N curves, the fatigue lives for various failure probabilities *P_f_* can be calculated by (4) [16,38].

(4) $$N=\ln^{-1}\left[\frac{\ln\left\{\ln\left(\frac{1}{1-Pf}\right)+\alpha \ln(u)\right\}}{\alpha }\right]$$

### 3.2. Three-Parameter Weibull Distribution Model 

Three-parameter Weibull distribution model involves the plotting of the cumulative distribution functions (CDFs) for fatigue lifetime at given stress range and stress range at given lifetime. Its elucidation delivers simply two possible solutions [35,42]:

(5) $$\mathrm{Model}\;I:\;F(N,\Delta \sigma )=1-\exp\left[{\left(\frac{\left(\log N-B\right)\left(\log\Delta \sigma -C\right)-\lambda }{\delta }\right)}^{\beta }\right];\left(\log N-B\right)\left(\log\Delta \sigma -C\right)\geq \lambda$$

(6) $$\mathrm{Model}\;\mathrm{II}:\;F(N,\Delta \sigma )=1-\exp\left[-{\left(\frac{\left(\log N-B\right){\left(\log\Delta \sigma -C\right)}^{\gamma }}{\delta }\right)}^{\beta }\right];\log N\geq B,\log\Delta \sigma \geq C$$

where, ‘*B*’ is a threshold value of the lifetime, ‘*C*’ is the endurance limit, or fatigue limit for *N* →∞, ‘*λ*’, ‘*δ*’, ‘*β*’ are location parameter, scale parameter, and shape parameter, respectively, whereas ‘*γ*’ is a parameter that scales the normalization of the S-N field.

To ease the applicability of this model for practical evaluation of the S-N field from fatigue data, the open source software program ProFatigue^®^ (Gijón-Asturias, España) was developed, by Fernandez-Canteli et al., in which the fatigue data can be easily modeled (Equations (5) and (6)). The model parameters are determined for appropriate fatigue data under consideration, thus permitting a probabilistic prediction of lifetime as a function of stress range for fixed stress level (R, σmean, etc.) [36,43].

## 4. Results and Discussion

Table 2 shows the resulting values of cycles to failure for various LCC specimens.

### 4.1. Two-Parameter Weibull Distribution Model

**Figure 2:**
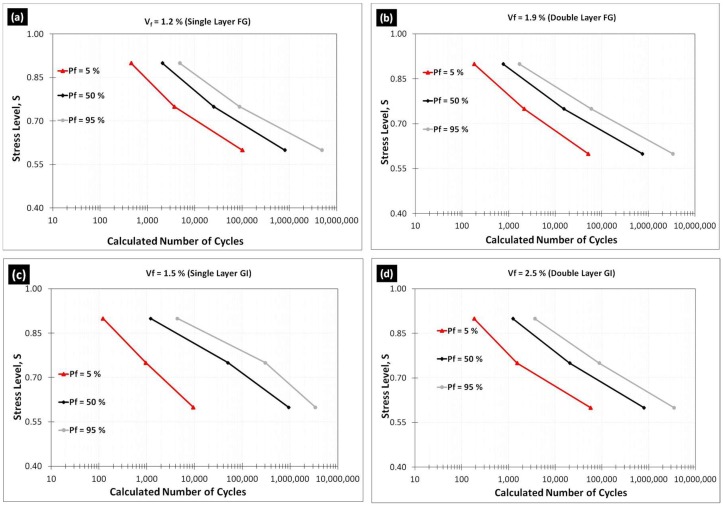
Probabilistic fatigue lives for various failure probabilities.

The probabilistic failure fatigue lives were analyzed using the two-parameter Weibull distribution model, and the results are plotted in Figure 2 indicating the probabilistic failure fatigue lives at different stress levels for varying failure probabilities. The details of calculations as well as the elucidation of applicability of the two-parameter model have already been covered earlier [25,27,28].

### 4.2. Three-Parameter Weibull Distribution Model

**Figure 3:**
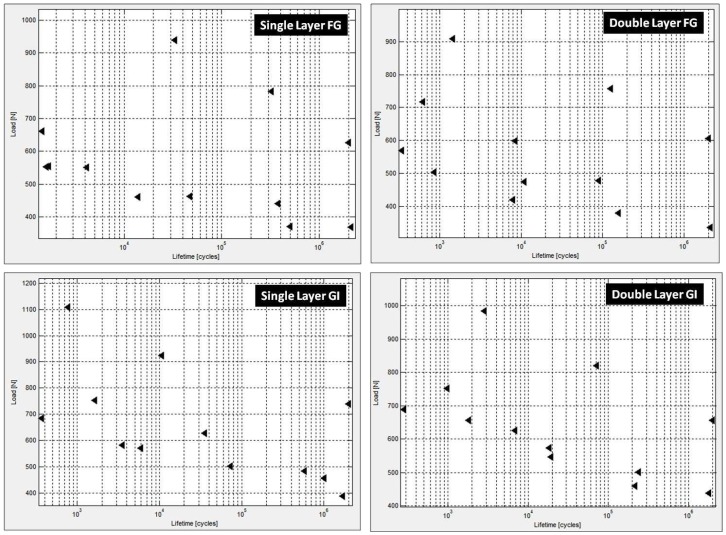
Load versus cycles to failure plots.

**Figure 4:**
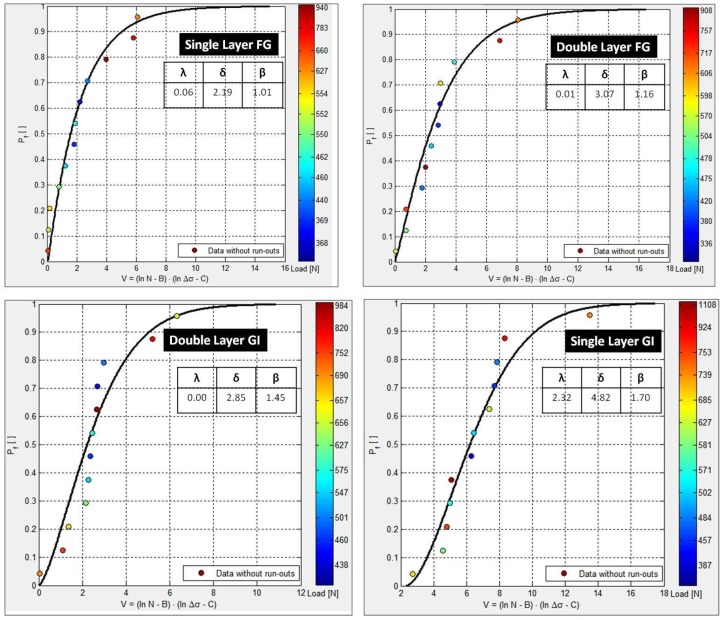
Weibull parameters and cumulative distribution function obtained by three-parameter Weibull distribution modeling.

**Figure 5:**
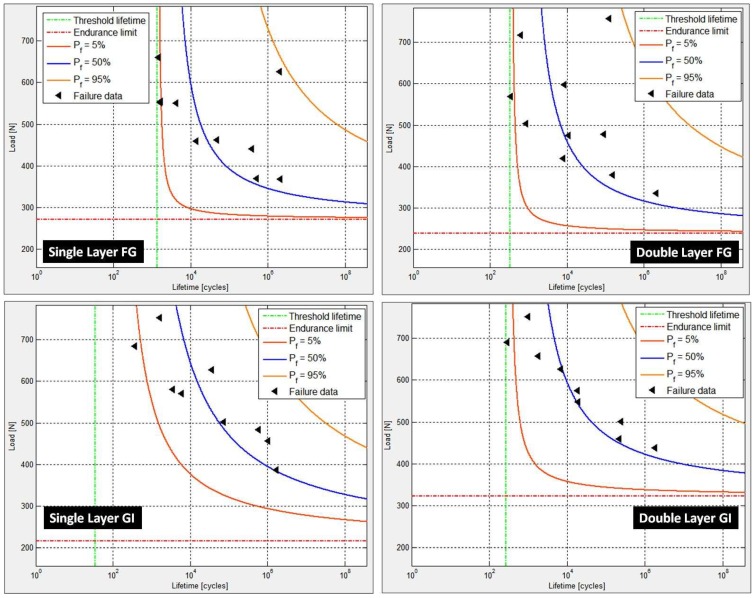
Probabilistic fatigue lives obtained through three-parameter Weibull distribution modeling.

The load versus fatigue life plotted in ProFatigue^®^ is shown in Figure 3 whereas the corresponding results—Weibull parameters and cumulative distribution function and probabilistic fatigue lives obtained by three-parameter Weibull distribution modeling—are shown in Figure 4 and Figure 5, respectively.

The absolute error was also determined which is not shown here to prevent unnecessary lengthy text. From the obtained results, it could be seen that the fatigue modeling of LCCs reinforced with single layer of mesh reinforcement exhibited more accurate results compared to double layer reinforced LCCs. This is evident from the better fitting of the CDF plot. Moreover, the location parameter ‘*λ*’ was found to be zero (shown in Figure 4) for LCC specimens reinforced with two layers of FG or GI mesh reinforcement. Thus, excluding one parameter (location parameter) corroborates that for such specimens (i.e., double layer reinforced LCCs), the three parameter model does not hold good, rather the two-parameter model is better suited.

It is pertinent to mention that the software is designed for isotropic materials, and uses material characteristics such as elastic modulus, elongation, Poisson’s ratio, etc. Although the matrix properties for FAC incorporated composites are experimentally determined, the greatly varying parameters (FAC weight fraction, water to binder ratio, reinforcement volume fraction) affect the resulting properties significantly. Moreover, the agglomeration issues with FACs yield high disparity in properties tested for various samples of the series.

## 5. Conclusions

The flexural fatigue of structural lightweight LCCs is critical for purposes, where cyclic loading is the governing factor in the failure of a particular structural member. In this study, lightweight LCCs containing FAC were tested under cyclic loading and the resulting fatigue data were used for carrying out probabilistic fatigue life analysis. The results corroborate that the flexural fatigue modeling for cement-based composites, particularly laminated composites, can be modeled by the double-parameter Weibull distribution model which can further be used to predict the failure life (number of cycles to failure when subjected to flexural cyclic loading). Moreover, the modeling approach adopted by ProFatigue^®^ (three-parameter Weibull model) is also adequate for statistical analysis of fatigue data corresponding to LCCs. However, the two-parameter model is more accurate for the probabilistic fatigue life assessment of double-layer reinforced LCCs, whereas for single-layer reinforced LCCs, both models could be used at a fair confidence level.

It is, however, pertinent to note that the fatigue in compression must also be evaluated in future to use the software-based method for fatigue life assessment. Use of ProFatigue^®^ can significantly reduce the computational effort and time.

## Figures and Tables

**Table 1:** Reinforcement properties [4,5,25].

Reinforcement Type	Manufacturer	Thickness (µm)	Opening (mm)	Yield Strength (Mpa)	Elastic Modulus (GPa)	Density (g/cm^3^)	Elongation (%)
Fiber Glass Mesh (FG)	HaoXin hardware mesh Co. Ltd., Wuxi, China	250	5 × 5	1100	75	2.6	3.9
Galvanized Iron Welded Wire Mesh (GI)	Xing Meng Zinc Steel Guardrail Co. Ltd., Chengdu, China	750	5 × 5	250	300	7.8	7
PVA Fibers	Kuraray Co., Ltd., Okayama, Japan	15	NA	1600	41	1.3	6

**Table 2:** Details of casted composites [25].

Series	Matrix	Mesh Type	No. of Layers	Mesh Volume Fraction, Vr (%)	PVA Fibers Volume Fraction, Vf (%)	Total Reinforcement Ratio, (%)
No. 1	Mix 1	GI	1	1.00%	0.50%	1.50%
No. 2	Mix 1	GI	2	2.00%	0.50%	2.50%
No. 3	Mix 1	FG	1	0.70%	0.50%	1.20%
No. 4	Mix 1	FG	2	1.40%	0.50%	1.90%
No. 5	Mix 2	GI	1	1.00%	0.50%	1.50%
No. 6	Mix 2	GI	2	2.00%	0.50%	2.50%
No. 7	Mix 2	FG	1	0.70%	0.50%	1.20%
No. 8	Mix 2	FG	2	1.40%	0.50%	1.90%
No. 9	Mix 3	GI	1	1.00%	0.50%	1.50%
No. 10	Mix 3	GI	2	2.00%	0.50%	2.50%
No. 11	Mix 3	FG	1	0.70%	0.50%	1.20%
No. 12	Mix 3	FG	2	1.40%	0.50%	1.90%
No. 13	Mix 4	GI	1	1.00%	0.50%	1.50%
No. 14	Mix 4	GI	2	2.00%	0.50%	2.50%
No. 15	Mix 4	FG	1	0.70%	0.50%	1.20%
No. 16	Mix 4	FG	2	1.40%	0.50%	1.90%

**Table 3:** S-N relationship for various thin laminated cementitious composites (LCCs).

Series	Cyclic Test (Number of Cycles, N)			Cyclic Test, log_10_(N)		
	Stress Range (0.10–0.60)	Stress Range (0.10–0.75)	Stress Range (0.10–0.90)	Stress Range (0.10–0.60)	Stress Range (0.10–0.75)	Stress Range (0.10–0.90)
Series 1	73,214	35,925	1646	4.86	4.56	3.22
Series 2	240,772	6916	975	5.38	3.84	2.99
Series 3	380,751	4144	1455	5.58	3.62	3.16
Series 4	89,960	8450	630	4.95	3.93	2.80
Series 5	1,016,836	5944	372	6.01	3.77	2.57
Series 6	214,647	18,446	283	5.33	4.27	2.45
Series 7	504,847	47,101	1695	5.70	4.67	3.23
Series 8	155,944	10,918	346	5.19	4.04	2.54
Series 9	1,703,252	584,742	3523	6.23	5.77	3.55
Series 10	1,808,915	19,458	1816	6.26	4.29	3.26
Series 11	2,112,929	14,022	1596	6.32	4.15	3.20
Series 12	2,097,152	7904	858	6.32	3.90	2.93
Series 13	2,000,000	10,649	773	6.30	4.03	2.89
Series 14	2,000,000	72,045	2867	6.30	4.86	3.46
Series 15	2,000,000	323,705	33,263	6.30	5.51	4.52
Series 16	2,000,000	126,156	1469	6.30	5.10	3.17

## References

[1] American Concrete Institute (2004). Report on Thin Reinforced Cementitious Products.

[2] Naaman A.E. (2000). Ferrocement and Laminated Cementitious Composites.

[3] Naaman A.E. (2002). Ferrocement: International Revival. ACI Spec. Publ..

[4] Hanif A., Cheng Y., Lu Z., Li Z. (2018). Mechanical Behavior of Thin-Laminated Cementitious Composites Incorporating Cenosphere Fillers. ACI Mater. J..

[5] Hanif A., Lu Z., Sun M., Parthasarathy P., Li Z. (2017). Green lightweight ferrocement incorporating fly ash cenosphere based fibrous mortar matrix. J. Clean. Prod..

[6] Yerramala A., Ramachandurdu C., Bhaskar Desai V. (2013). Flexural strength of metakaolin ferrocement. Compos. Part B Eng..

[7] Memon N.A., Sumadi S.R., Ramli M. (2007). Performance of high wokability slag-cement mortar for ferrocement. Build. Environ..

[8] Desayi P., Reddy V. (1991). Strength of Lightweight Ferrocement in Flexure. Cem. Concr. Compos..

[9] Memon N.A., Sumadi S.R., Ramli M. (2007). Ferrocement encased lightweight aerated concrete: A novel approach to produce sandwich composite. Mater. Lett..

[10] Hanif A. (2017). Development and Application of High Performance Lightweight Cementitious Composite for Wind Energy Harvesting.

[11] Paskova T., Meyer C. (1997). Low-cycle Fatigue of Plain and Fiber-Reinforced Concrete. ACI Mater. J..

[12] Hsu T.T.C. (1981). Fatigue of Plain Concrete. ACI J..

[13] Tepfers R., Hedberg B., Szczekocki G. (1984). Absorption of energy in fatigue loading of plain concrete. Mateiraux Constr..

[14] Byung H.O. (1986). Fatigue Analysis of Plain Concrete in Flexure. ASCE J. Struct. Eng..

[15] Naik T.R., Singh S.S., Ye C. (2015). Fatigue Behavior of Plain Concrete Made with or Without Fly Ash.

[16] Singh S.R., Kaushik S.K. (2000). Flexural fatigue life distributions and failure probability of steel fibrous concrete. ACI Struct. J..

[17] Saleh M.F., Yeow T., Macrae G., Scott A. (2012). Effect of steel fibre content on the fatigue behaviour of steel fibre reinforced concrete. RILEM Bookseries.

[18] Yan H., Sun W., Chen H. (1999). The effect of silica fume and steel fiber on the dynamic mechanical performance of high-strength concrete. Cem. Concr. Res..

[19] Behbahani H.P. (2010). Flexural Behavior of Steel Fibers Reinforced Concrete Beams. Master’s Thesis.

[20] Chang D.I., Chai W.K. (1995). Flexural fracture and fatigue behavior of steel-fiber-reinforced concrete structures. Nucl. Eng. Des..

[21] Batson B.G., Ball C., Bailey L., Landers E., Hooks J. (1972). Flexural Fatigue Strength of Steel Fiber Reinforced Concrete Beams. J. Proc..

[22] Jang J.G., Kim H.K., Kim T.S., Min B.J., Lee H.K. (2014). Improved flexural fatigue resistance of PVA fiber-reinforced concrete subjected to freezing and thawing cycles. Constr. Build. Mater..

[23] Ranjbarian M., Mechtcherine V. (2018). A novel test setup for the characterization of bridging behaviour of single microfibres embedded in a mineral-based matrix. Cem. Concr. Compos..

[24] Hanif A., Kim Y., Parthasarathy P., Usman M., Li Z. Flexural Fatigue Behavior of Lightweight Ferrocement: Experimental Investigation & Numerical Modeling. Proceedings of the International Federation for Structural Concrete 5th International FIB Congress 2018.

[25] Hanif A., Usman M., Lu Z., Cheng Y., Li Z. (2018). Flexural Fatigue Behaviour of Thin Laminated Cementitious Composites Incorporating Cenosphere Fillers. Mater. Des..

[26] Huang B., Li Q., Xu S., Zhou B. Investigation on Compressive Fatigue Damage Process of Ultra-High Toughness Cementitious Composites. Proceedings of the 9th International Conference on Fracture Mechanics of Concrete and Concrete Structures.

[27] Huang B.-T., Li Q.-H., Xu S.-L., Liu W., Wang H.-T. (2018). Fatigue deformation behavior and fiber failure mechanism of ultra-high toughness cementitious composites in compression. Mater. Des..

[28] Huang B.-T., Li Q.-H., Xu S.-L., Zhou B.-M. (2018). Tensile fatigue behavior of fiber-reinforced cementitious material with high ductility: Experimental study and novel *P-S-N* model. Constr. Build. Mater..

[29] Huang B.-T., Li Q.-H., Xu S.-L., Zhou B.-M. (2017). Frequency Effect on the Compressive Fatigue Behavior of Ultrahigh Toughness Cementitious Composites: Experimental Study and Probabilistic Analysis. J. Struct. Eng..

[30] Huang B.-T., Li Q.-H., Xu S.-L. (2019). Fatigue Deformation Model of Plain and Fiber-Reinforced Concrete Based on Weibull Function. J. Struct. Eng..

[31] Singh S.P., Mohammadi Y., Kaushik S.K. (2005). Flexural Fatigue Analysis of Steel Fibrous Concrete Containing Mixed Fibers. ACI Mater. J..

[32] Behloul M., Chanvillard G., Pimienta P., Pineaud A., Rivillon P. Fatigue Flexural Behavior of Pre-cracked Specimens of Special UHPFRC. Proceedings of the Seventh International Symposium on the Utilization of High Strength/High-Performance Concrete.

[33] Mohamadi M.R., Mohandesi J.A., Homayonifar M. (2013). Fatigue behavior of polypropylene fiber reinforced concrete under constant and variable amplitude loading. J. Compos. Mater..

[34] Castillo E., Ramos A., Koller R., López-Aenlle M., Fernández-Canteli A. (2008). A critical comparison of two models for assessment of fatigue data. Int. J. Fatigue.

[35] Castillo E., Fernendez-Canteli A. (2009). A Unified Statistical Methodology for Modeling Fatigue Damage.

[36] Fernández-Canteli A., Przybilla C., Nogal M., Aenlle M.L., Castillo E. (2014). ProFatigue: A Software Program for Probabilistic Assessment of Experimental Fatigue Data Sets. Procedia Eng..

[37] Hanif A., Parthasarathy P., Ma H., Fan T., Li Z. (2017). Properties Improvement of Fly Ash Cenosphere Modified Cement Pastes Using Nano-Silica. Cem. Concr. Compos..

[38] Goel S., Singh S.P., Singh P. (2013). Fatigue Analysis of Plain and Fiber-Reinforced Self Consolidating Concrete. ACI Mater. J..

[39] Oh B.H. (1991). Fatigue Life Distributions of Concrete for Various Stress Levels. ACI Mater. J..

[40] Singh S.P., Mohammadi Y., Madan S.K. (2006). Flexural fatigue analysis of steel fibrous concrete containing mixed fibers. J. Zhejiang Univ. Sci. A.

[41] Singh S.P., Kaushik S.K. (2003). Fatigue strength of steel fibre reinforced concrete in flexure. Cem. Concr. Compos..

[42] Przybilla C., Fernández-Canteli A., Castillo E. (2013). Maximum likelihood estimation for the three-parameter Weibull cdf of strength in presence of concurrent flaw populations. J. Eur. Ceram. Soc..

[43] Pyttel B., Canteli A.F., Ripoll A.A. (2016). Comparison of different statistical models for description of fatigue including very high cycle fatigue. Int. J. Fatigue.

